# Medicinal Plants and Fungi Traditionally Used by Dulong People in Northwest Yunnan, China

**DOI:** 10.3389/fphar.2022.895129

**Published:** 2022-05-09

**Authors:** Zhuo Cheng, Xian Hu, Xiaoping Lu, Qiong Fang, Yuan Meng, Chunlin Long

**Affiliations:** ^1^ Key Laboratory of Ecology and Environment in Minority Areas (Minzu University of China), National Ethnic Affairs Commission of China, Beijing, China; ^2^ College of Life and Environmental Sciences, Minzu University of China, Beijing, China; ^3^ Key Laboratory of Ethnomedicine (Minzu University of China), Ministry of Education, Beijing, China

**Keywords:** Dulong, traditional knowledge, ethnobotany, medicinal plants, Dulongjiang region

## Abstract

The Dulong, an ethnic group living in the isolated Northwest Yunnan of Southwest China, have directly used a wide of plants to serve their needs and have accumulated rich traditional knowledge about medicinal plants over years. Unfortunately, little has been reported about the medicinal plants used by the Dulong people. Ethnobotanical data were collected through semi-structured interviews, guided field trips, and quantitative analysis. Prior informed consent was obtained before each interview. The surveys allowed for the collection of sociodemographic data and traditional knowledge about medicinal plants and their uses. This study used relative frequency of citation (RFC) to identify the most culturally significant medicinal plants and used informant consensus factor (FIC) to evaluate agreement among informants. A total of 105 medicinal plant species belonging to 69 families were recorded. Amongst these 69 families, Asteraceae (8 species), Polygonaceae, Ranunculaceae, and Rosaceae (4 species each) were the dominant families. The whole plants were the most frequently used part in the preparation of medicines. The most common preparation method was decoction and the most frequent application route was oral administration. *Coptis teeta* (0.15), *Acorus calamus* (0.12), *Ophiocordyceps sinensis* (0.11), *Tanacetum tatsienense* var. *tanacetopsis* (0.11), and *Paris polyphylla* var. *yunnanensis* (0.08) were shown to be the most useful plants as indicated by their relatively high RFC values. Among the usage types of medicinal plants, the highest FIC values were recorded for the circulatory system (FIC = 0.91), the immune system (FIC = 0.89), and the nervous system (FIC = 0.85). Furthermore, sixty-two medicinal plants utilized by the Dulong for medicinal purposes also have dietary use. Traditional knowledge associated with medicinal plants has been seriously threatened in recent decades. In the future, modern approaches should be used to demystify traditional medicine. However, significant measures need to be taken to protect from loss the important traditional knowledge gained by the Dulong through their experience and inheritance. A collective effort should be made to promote and conserve the important traditional medicinal knowledge and outline a plan for sustainable use of medicinal plants and improve local economic development under the premise of protection.

## 1 Introduction

Since ancient times, humans have established a close relationship with plants. The traditional knowledge of plants used for medicines, foods, fibers, building materials, cosmetics, dyes, agrochemicals, fuel, religion, and ritual purpose was generated and accumulated in the reciprocal interactions between humans and plants, among which traditional knowledge of medicinal plants especially plays an irreplaceable role in human life (Schmidt and Cheng, 2017). As an important source of medicine, plants were cited around the globe as cures to almost all disease categories (Rahman et al., 2018). For remote areas in developing countries, traditional knowledge of medicinal plants plays a vital role in safeguarding the health and promoting the economic prosperity of local people (Pardo-de-Santayana and Macía, 2015; Quave and Pieroni, 2015; Li and Weng, 2017). At the same time, the traditional knowledge of these medicinal plants is also important for new drug development with potential to export and import of diverse parts or bioactive compounds in the market (Samy and Gopalakrishnakone, 2007; Ali et al., 2021).

China is a country with a long history, rich biodiversity, and diverse ethnic cultures (Mi et al., 2021). There are approximately 11,000 species of medicinal plants in China (Pei and Huai, 2007; Zhang and Yang, 2012). Over the long history and development of different linguistic groups, they have accumulated traditional knowledge of using medicinal plants to treat diseases and to resist the harsh natural environment. On this basis, they have created colorful ethnomedicines (Pei, 2001). However, with the infiltration of mainstream cultures, the destruction of the natural environment, and the expansion of urbanization, traditional medicinal knowledge is facing the danger of assimilation and loss (Luo et al., 2018). Therefore, traditional knowledge of medicinal plants needs to be recorded for its protection without any further delays (Cox, 2000; Yao et al., 2021; Huang et al., 2022). A comprehensive study should be carried out on the traditional knowledge of medicinal plants. The endangered traditional knowledge should be classified and recorded, which will help promote regional economic development and ensure the protection and sustainable use of medicinal plants (Cheng et al., 2020a).

Traditional medicinal knowledge results from adapting to the living environment (Geng et al., 2020; Gras et al., 2021). With the economic development of the Dulongjiang region, traditional medicinal plants are inevitably subjected to varying degrees of impact in the modernization process. It is imperative to record, categorize, research, and inherit the essence of traditional medicinal plants (Cheng et al., 2020a).

Due to the isolation of the Dulongjiang region, only a few studies have been reported involving medicinal plants (Long et al., 1999; Li, 2012). These studies have not systematically and comprehensively investigated the traditional medicinal knowledge of the Dulong. In particular, there is a lack of evaluation methods using quantitative indices (Sun et al., 2011). Therefore, the aims of this study are: 1) Record and organize the traditional medicinal plants of the Dulong; 2) Evaluate the consistency of usage of traditional medicines of the Dulong in the treatment of diseases; 3) Analyze the threats to the traditional medicine of the Dulong; 4) Protect the knowledge associated with this traditional medicine so that it can better serve the health and economy of the Dulong.

## 2 Material and Methods

### 2.1 Study Area

**FIGURE 1:**
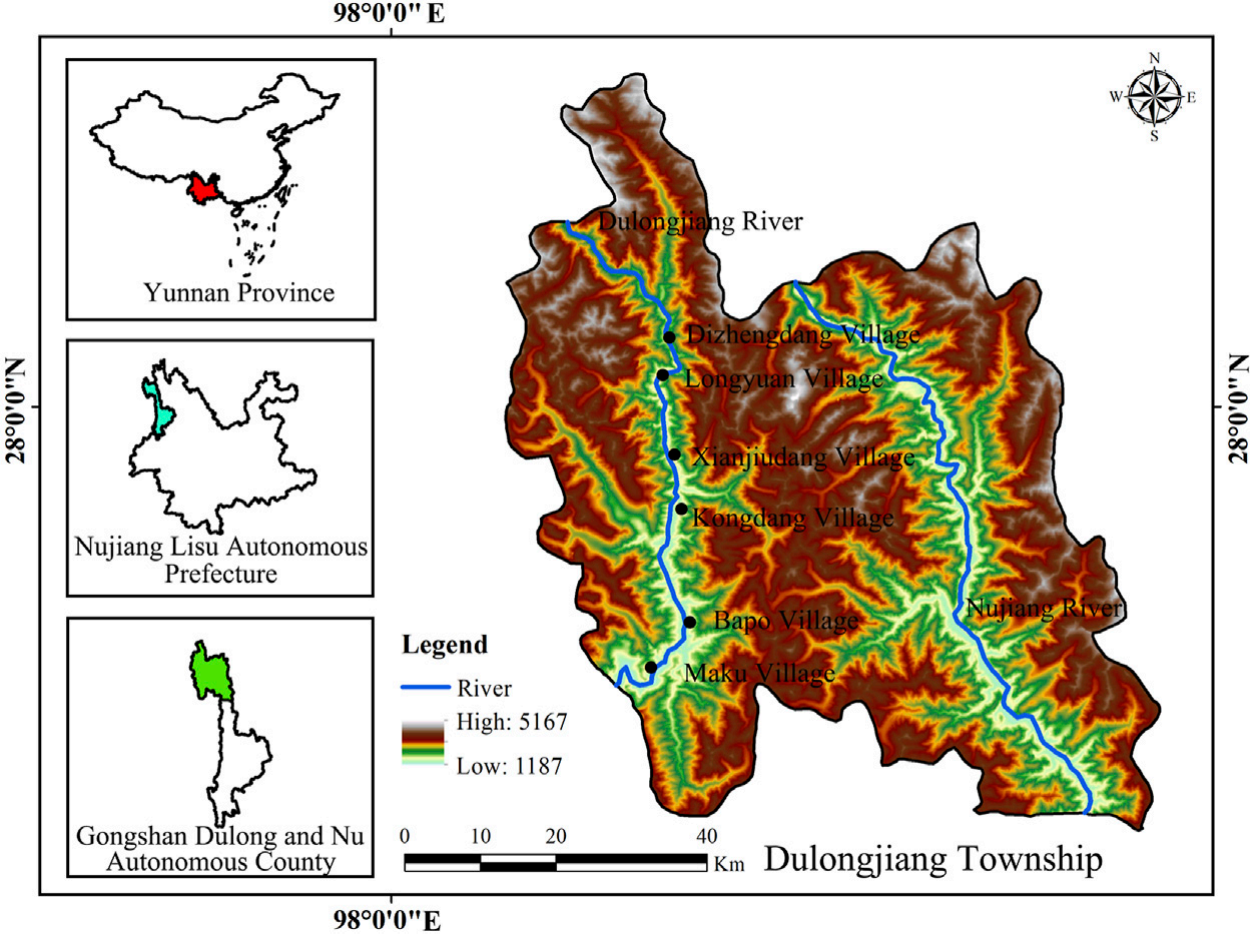
Study area.

Dulongjiang Township is located in the northwest of Yunnan Province, China (27°40′ to 28°50′ N, 97°45′ to 98°30″ E), with a total area of 1,994 km^2^. From the upper reaches to the lower reaches of the Dulongjiang River are Dizhengdang Village, Longyuan Village, Xianjiudang Village, Kongdang Village, Bapo Village and Maku Village (Figure 1). Dulongjiang Township is the only settlement of Dulong people in China, with special alpine and gorge landforms, diverse climates, closed traffic conditions, rich biological resources, self-sufficient social economy and unique national culture. Dulongjiang Township is also one of the most biodiverse areas in China, with more than 2,000 species of seed plants in the area. Among them, there are 940 species of economic plants, and there are more than 500 species of plants with medicinal value, such as *Paris polyphylla* var. *yunnanensis*, *Coptis teeta*, *Houpoea rostrata*, *Gastrodia elata*, *Polygonatum cirrhifolium*, *Tuber melanosporum*, and *Cordyceps sinensis*.

Dulong, a cross-boundary ethnic group in China, with a total population of 6,930 people, mainly live in Dulongjiang area. It is a township in Gongshan Dulong and Nu Autonomous County, Nujiang Lisu Autonomous Prefecture, Yunnan Province (Li, 1994). The area is known as the “the last secret place in southwest China” (Li et al., 2011). For a long time, the high and majestic Gaoligongshan has blocked the Dulong from interacting with the outside world (Li, 1994). In the process of long-term mutual adaptation with the living environment, they rely on plants to serve their survival needs and maintain life (Geng et al., 2017). For example, there is the collection of wild edible plants, medicinal plants, traditional beekeeping, cultivation of *Caryota obtusa*, and fuelwood collection (Zhou et al., 2011; Li, 2012; Cheng et al., 2020a, 2022).

### 2.2 Field Survey and Data Collection

The field survey was conducted four times from 2019 to 2020, and the total survey lasted for about 3 months. The survey sites included six villages. Field investigations include semi-structured interviews and participatory observation. A total of 155 informants in the Dulongjiang region were interviewed. Informed consent was obtained verbally from all participants prior to the study (International Society of Ethnobiology, 2006).

During the field investigations, the informants were invited to list all medicinal plants that are still in use or have been used. The interview consisted of two parts: the first part was about the basic information of the informant (ethnic group, gender, age, education, occupation), and the other part included questions related to the detailed information of all medicinal plants, including the locality of the reported medicinal plants, names, use parts, processing methods, application methods, and medicinal effects. The data was recorded and quantitatively analyzed using different quantitative indices.

The nomenclature of all vascular plants follows the *Plants of the World Online* (https://powo.science.kew.org/) and the voucher specimens were deposited at the herbarium of the ethnobotany laboratory of the College of Life and Environmental Sciences, Minzu University of China in Beijing.

### 2.3 Data Analysis and Quantitative Indexes

The ethnobotanical data were recorded and analyzed using Microsoft Office Excel 2016, and Origin 2018 was used for drawing graphs and images. The categories suggested for diseases were classified according to International Classification of Primary Care (ICPC) (https://www.who.int/classifications/icd/en/), system for intercultural comparisons, with minor modifications (Staub et al., 2015).

Relative Frequency of Citation: $\text{RFC}=\frac{\mathrm{FC}}{N}$

The index shows the local importance of medicinal species by frequency of citation (FC) (Barkaoui et al., 2017; Al-Fatimi, 2019). The frequency of citation divided by the number of all informants participating in the survey (N). The higher the RFC, the more important the medicinal plant is in the area (Tardío and Pardo-de-Santayana, 2008).

Informant Consensus Factor: $\text{FIC}=\frac{\mathrm{Nur}-\mathrm{Nt}}{\mathrm{Nur}-1}$

FIC analyzes the degree of difference in the types of medicinal plants used by different herbalists to treat a certain type of disease. It can also reflect the importance of certain plants in the treatment of a certain type of disease in a specific cultural group.

In the formula, Nur is the number of using reports in a particular category and Nt is the number of plant species recorded in the category. FIC values range from 0 to 1. Values close to 1 indicate that relatively few taxa are used by a large number of healers, or there is a clear standard for the use of specific plant species in the community. A low value (close to 0) indicates that the informant does not agree with the use of the plant species in the above treatment categories (Trotter and Logan, 1986).

## 3 Results

### 3.1 Demographic Features of the Respondents

A total of 155 informants from six villages were interviewed, including 101 males (65.2%) and 54 females (34.8%). The informants ranged in age from 14 to 95, and the mean age was 42, with most of them in the groups between 20 and 39 years old (45.8%), and between 40 and 59 (34.8%). All informants are the Dulong, and the formal education level of the interviewees was low. Among them, 19.4% were illiterate, 25.2% with primary education, and 39.4% with middle high school education. The majority of informants interviewed were engaged in farming work (77.4%), followed by salaried temporary work and trading, and a few were students (Table 1).

**TABLE 1:** Demographic characteristic of respondents.

Characteristics		Frequency	Percentage (%)
Gender	Male	101	65.2
	Female	54	34.8
Age	Less than 20	6	3.9
	20–39	71	45.8
	40–59	54	34.8
	More than 59	24	15.5
Formal education	Illiterate	30	19.4
	Primary school	39	25.2
	Middle high school	61	39.3
	High school	25	16.1
Main occupation	Farming	120	77.4
	Salaried temporary work	26	16.8
	Trading	4	2.6
	Student	5	3.2

### 3.2 Diversity of Medicinal Plants

**FIGURE 2:**
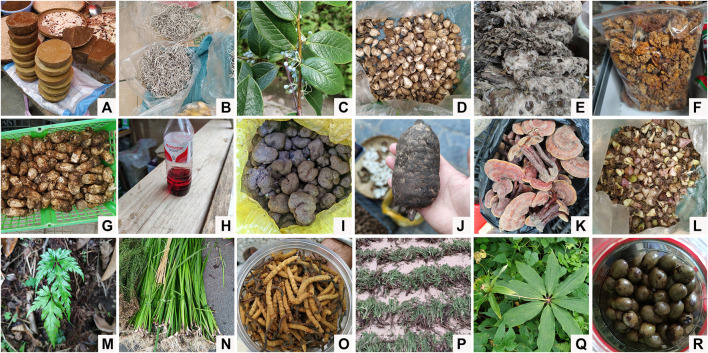
Some medicinal plants with high RFC. **(A)** The fat extracted from *Toxicodendron vernicifluum*; **(B)** The whole plant of *Thamnolia vermicularia*; **(C)** The fruit of *Vaccinium gaultheriifolium*; **(D)** The bulb of *Fritillaria cirrhosa*; **(E)** the whole plant of *Saussurea obvallata*; **(F)** The fruit body of *Tremella aurantialba*; **(G)** The fruit body of *Tricholoma matsutake*; **(H)** The fruit body of *Hypocrella bambusae*; **(I)** The fruit body of *Tuber melanosporum*; **(J)** The rhizome of *Gastrodia elata*; **(K)** The fruit body of *Ganoderma lucidum*; **(L)** The rhizome of *Bletilla striata*; **(M)** the whole plant of *Coptis teeta*; **(N)** The whole plant of *Acorus calamus*; **(O)** The fruit body of *Ophiocordyceps sinensis*; **(P)** The whole plant of *Tanacetum tatsienense* var. *tanacetopsis*; **(Q)** The whole plant of *Paris polyphylla* var. *yunnanensis*; **(R)** The fruit of *Elaeocarpus lacunosus*.

The medicinal plants used by the Dulong are diverse. The 155 informants reported a total of 105 taxa including 95 vascular plant species, 8 fungi species, and 2 lichen species used by the Dulong for curative purposes. The Dulong use multifarious types of medicinal plants, including ferns, gymnosperms, angiosperms, fungi, and lichens (Figure 2). All above groups belong to plant *sensu lato* (kingdom Plantae) according to the two-kingdom system. Botanical information (scientific names, vernacular names, families, and specimen numbers) and ethnobotanical information (medicinal parts, medicinal effect, modes of preparations, and administration) of all plants are listed in Supplementary Table S1.

#### 3.2.1 Family Distribution of Medicinal Plants

A total of 105 medicinal plant species belonging to 69 families were recorded. The plant species recorded in the study area were presented in Supplementary Table S1, arranged in alphabetical order for families and entities. The most frequently used families were Asteraceae (8 species), Polygonaceae, Ranunculaceae, and Rosaceae (4 species each), Apiaceae, Lauraceae, Liliaceae, and Poaceae (3 species each), and were among the dominant families. The remaining families were represented by 2 or fewer entities. In previous studies, these families were also reported to be widely used by ethnic minorities in northwest Yunnan, China (Liu et al., 2016).

#### 3.2.2 Used Parts of Medicinal Plants

The Dulong used various parts of medicinal plants, including 11 types of organized parts and 2 types of unorganized parts (exudates products). For example, the Dulong used the turpentine of *Pinus yunnanensis* to treat traumatic injury and used the latex of *Nabalus tatarinowii* to stop bleeding. The most frequently used plant parts were the whole plant, followed by rhizome, root, leaf, stem, fruit, fruit body, tuber, bark, bulb, and seed. Studies have shown that the use of whole plants, rhizomes, and roots was not sustainable for the use of medicinal plants (Liu et al., 2016).

#### 3.2.3 Modes of Preparation and Administration

Five different modes of medicine preparation and four different modes of medicine application were documented. Decoction was the most commonly used processing method, followed by crushed, cooked (fried, roasted, stewed, sliced), alcohol maceration, and processing into powder or oil. A total of two routes of administration were quoted by the Dulong. Oral administration was the most frequently used route, followed by external application (Table 2).

**TABLE 2:** Mode of preparation and administration of medicinal plants.

Mode of preparation	Numbers	Percentage (%)	Application method	Numbers	Percentage (%)
Decoction	49	42.2	Oral	74	70.5
Crushed	30	25.9	External application	31	29.5
Cooked (fried, toasted, stewed, sliced)	22	19.0			
Alcohol maceration	8	6.9			
Processed into powder or oil	7	6.0			

### 3.3 Relative Frequency of Citation of Medicinal Plants

The RFC can reflect the importance of medicinal plants in the community. *Coptis teeta* (0.15), *Acorus calamus* (0.12), *Ophiocordyceps sinensis* (0.11), *Tanacetum tatsienense* var. *tanacetopsis* (0.11), and *Paris polyphylla* var. *yunnanensis* (0.08) were shown to be the most useful plants as indicated by their relatively high RFC (Figures 2M–R).

With a wide range of pharmacological activities, like anti-tumor, antimicrobial, and anti-inflammatory, *C. teeta* is one of the famous regional drug source in northwest Yunnan (Huang and Long, 2006, 2007). The main components of *C. teeta* are alkaloids such as berberine, jateorhizine, and palmatine. Studies have shown that the berberine content of *C. teeta* is higher than other plants of the same genus (Yang et al., 2011). Due to habitat loss, change of land use type, and over-collection, the population of *C. teeta* has declined sharply and is now endangered. At present, *C. teeta* is listed as critically endangered on ICUN Red List. In the past, the collection of *C. teeta* was one of the main economic of sources of the local ethnic groups like Lisu, Nu, and Dulong (Huang and Long, 2007). In the 1950s, the sale of *C. teeta* brought in 50% of their total revenue. Studies have shown that these ethnic groups previously used *C. teeta* sustainably, based on their religion, including worship of *C. teeta* planted forests, through the “customary law” to regulate the planting and management of *C. teeta*, and formed an of effective, scientifically significance based system that mixed agriculture, and forestry in a sustainable system (Huang and Long, 2006).

As an important cultural species, *Acorus calamus* (*chang pu* in Chinese) is often used on the Dragon Boat Festival (Shu et al., 2018). During the Dragon Boat Festival, people often hang *A. calamus* and *Artemisia argyi* (*ai* in Chinese) together on the front door to protect family members from evil. Different ethnic groups use *A. calamus* for different purposes, such as cleaning water, repelling mosquitoes and wrapping *zongzi* (Shu et al., 2018; Lin et al., 2019). The majority of the Dulong will grow *A. calamus* in their home gardens to get a ready sufficiently of the medicine, and for ornamental and cultural value. Dulong regarded *A. calamus* as a sacred and symbolic plant because the leaves of the *A. calamus* look like swords, with a believed supernatural power to exorcise evil spirits. Local people believe that the *A. calamus* protects them from diseases. They would place necklaces with *A. calamus* around children’s necks, believing that this would prevent them from getting sick (Cheng et al., 2020b).

*Ophiocordyceps sinensis*, *Tanacetum tatsienense* var. *tanacetopsis*, and *Paris polyphylla* var. *yunnanensis* are important medicinal plants and cash income sources often collected and used by the Dulong. It is very easy to see these medicinal plants in local markets. The Dulong collect *O. sinensis* every year around April or May, and each one sells for about 5–10 RMB. *T. tatsienense* var. *tanacetopsis* is a traditional medicine of the Dulong, and its local name is “Mu Qiu”. It can be taken orally by decocting in hot water to treat indigestion and rheumatism. The Dulongjiang area is very rich in wild resources of *Paris*. There were three *Paris* species (*P. dulongensis*, *P. forrestii*, and *P. polyphylla* var. *yunnanensis*) cultivated by the local people. Due to the appropriate growing environment and strong supporting policy, the plantation of *P. polyphylla* var. *yunnanensis* has become one of the main income sources of the Dulong in recent years.

### 3.4 Informant Consensus Factor

Informant consensus factor can analyze the difference of medicinal plant species used by local people in the treatment of a certain type of disease. In a specific cultural group, it can objectively reflect the importance of certain plants in the treatment of a certain type of disease (Miara et al., 2018; Ojha et al., 2020). Fifty kinds of diseases reported by the informant were divided into 12 categories. The FIC values for all disease types ranged from 0.5 to 0.91 (Table 3). The type of disease with highest in FIC was the circulatory system (0.91), followed by the immune system (0.89), nervous system (0.85), digestive system (0.77), injury (0.75), and musculoskeletal system (0.75). The high value of FIC for circulatory, immune, and nervous system diseases may be due to the limited number of reports and information. The Nt and Nur of digestive system (22, 92), injury (22, 86), and musculoskeletal system (21, 81) were all relatively high, indicating that the Dulong had high consistency in the treatment of diseases of the digestive system, injury, and musculoskeletal system.

**TABLE 3:** Informant consensus factor by categories of diseases in the study area.

Category	Specific conditions	Nt	Nur	FIC
Infectious disease	Dysentery (9), hepatitis (6), malaria (3), pneumonia (8), phthisis (2)	10	28	0.67
Nervous system disease	Neurasthenia (4), insomnia (10)	3	14	0.85
Immune system disease	Tumour (3), hypoimmunity (10), intoxication (2), Deficiency of the kidney (23)	5	38	0.89
Ear and fever disease	Tinnitus (5), headache (17), mouth sores (3), fever (1)	8	26	0.72
Circulatory system disease	Heart disease (20), anemia (4)	3	24	0.91
Respiratory system disease	Cold (31), cough (29), bronchitis (3), pertussis (3), amygdalitis (11), pharyngalgia (3), asthma (5)	25	85	0.71
Digestive system disease	Gastritis (16), stomachache (17), dyspepsia (29), diarrhea (25), acute cholecystitis (1), jaundice (4)	22	92	0.77
Skin disease	Skin itch (4), skin ulcer (1), skin allergy (1)	6	11	0.50
Musculoskeletal system disease	Rheumatism (36), rheumatic arthritis (13), fracture (22), arthralgia (5), backache (5)	21	81	0.75
Genitourinary system disease	Urinary infection (5), nephritis (2)	4	7	0.50
Gynecological aliments	Postpartum recovery (5), irregular menstruation (1), gynaecopathia (5), leucorrhea (4)	5	15	0.71
Injury	Animal bite (18), traumatic injury (15), bleeding (5),wound (21), cut (15), burn (2)	22	86	0.75

### 3.5 Conservation Status

The conservation status of all recorded plant species was checked using the International Union for Conservation of Nature (IUCN) Red List of Threatened Species, Information System of Chinese Rare and Endangered Plants (http://www.iplant.cn/rep/), Convention on International Trade in Endangered Species of Wild Fauna and Flora (https://cites.org/eng) (IUCN, 2021). There are 32 medicinal plant species used by the Dulong that can be founded in the IUCN list, and six species are in threatened status (Supplementary Table S1). *Coptis teeta* and *Magnolia rostrata* were recorded as endangered (EN), and four species (*Gastrodia elata*, *Paris polyphylla* var. *yunnanensis*, *Ophiocordyceps sinensis*, *Tricholoma matsutake*) were recorded as vulnerable (VU). Besides, *Dendrobium nobile*, *Bletilla striata*, *Caryota obtusa*, *Cibotium barometz* and *Fagopyrum dibotrys* belong to the national key preserved wild plants (NKPWP) in China (http://www.iplant.cn/rep/). In particular, *D. nobile* is a first-class national key preserved wild plant. In addition, among these protected endangered plants, three species (*B. striata*, *C. barometz*, and *G. elata*) are listed in the appendix of CITES (Table 4). Although these species are under no threat of extinction, they still face trade pressure and are subject to international trade control.

**TABLE 4:** Rare and endangered medicinal plants collected by the local people.

Species	NKPWP	IUCN	CITES
*Dendrobium nobile* Lindl.	(I)		
*Bletilla striata* (Thunb.) Reichb.f.	(II)		II
*Caryota obtusa* Griff.	(II)	LC	
*Cibotium barometz* (L.) J. Sm.	(II)		II
*Coptis teeta* Wall.	(II)	EN	
*Fagopyrum dibotrys* (Lehm.) Mansf. ex K.Hammer	(II)		
*Gastrodia elata* Blume	(II)	VU	II
*Magnolia rostrata* W.W.Sm.	(II)	EN	
*Paris polyphylla* var. *yunnanensis* (Franch.) Hand.-Mazz.	(II)	VU	
*Ophiocordyceps sinensis* (BerK.) Sacc.		VU	
*Tricholoma matsutake* (Ito et Imai) Singer		VU	

### 3.6 Comparison of Traditional Medicine Used by Dulong People and in the Prefecture

*Traditional Medicine of Nujiang Region* (TMNR) is a book about medicinal plants used by ethnic minorities in the Nujiang Prefecture (Zhou and Zheng, 2010). The book recorded 189 medicinal plant species used by ethnic minorities (Lisu, Nu, Dulong and Pumi). In this study, 105 traditional medicinal plant species used by Dulong people were recorded. Comparing with the book, 45 overlapped species between the traditional medicine of Dulong people and the traditional medicine of Nujiang Prefecture, accounting for 42.9% of the total number of the traditional medicine of Dulong people. There are 60 medicinal plants that have not been recorded in TMNR.

### 3.7 New Reports and New Uses

By comparing the recorded medicinal plants from this study with other research and databases, we found that four species have not been previously recorded as medicinal plants. *Elaeocarpus lacunosus* and *Vaccinium gaultheriifolium* are medicinal dietary plants. The fruit of *E. lacunosus* was used by the Dulong people to treat traumatic injury. Studies have shown that the fruit of *E. ganitrus* could act as opioid receptor antagonists to reduce the secretion of neurotransmitters such as acetylcholine, norepinephrine, and dopamine, showing good analgesic effects (Katavic et al., 2007; Hong et al., 2019; Prasannan et al., 2020). The Dulong people use the fruit of *Vaccinium gaultheriifolium* to treat rheumatism. Studies have shown that the fruits of *Vaccinium* species represent an important natural source of antioxidants, such as polyphenolic compounds (anthocyanins, flavonols, phenolic acids, and proanthocyanidins) and ascorbic acid that attributed to antioxidant properties (Jurikova et al., 2018). Dulong people used *Selaginella sinensis* to treat heart diseases. *Selaginella* species are used in traditional medicine for the treatment of various diseases and conditions in Asia (Bailly, 2021). *S. plana* were also recorded to treat heart diseases (Dwi, 2009). In China, *S. tamariscina* is documented in Chinese Pharmacopoeia and used as a traditional medicine for promoting blood circulation. The extract of *S. tamariscina* was found to have distinctive vasorelaxant activity (Kang et al., 2004). The chemical constituents and pharmacological studies of species in the genus *Nabalus* have not been reported up to now. Further scientific verifications are needed to reveal their pharmacological mechanisms. In the present study, we also reported 19 new therapeutic uses for 17 known medicinal plant species. These new uses compared to those previously reported in other research and databases were described in Table 5.

**TABLE 5:** List of new therapeutic uses recorded in Dulongjiang area.

Botanical name	Part used	New uses	Previously reported uses	References
*Saurauia napaulensis* DC.	Root	Traumatic injury	Fever and viral diseases	Ozukum et al. (2019)
*Viburnum cylindricum* Buch.-Ham. ex D. Don	Leaf	Cold	Dysentery, acute gastroenteritis, stomatitis, urinary tract infection, burns, cutaneous pruritus	Yang et al. (2016); Chi et al. (1991)
*Allium hookeri* Thwaites	Whole plant	Traumatic injury	Relieve swelling and pain	Zhu (1991)
*Maianthemum atropurpureum* (Franch.) LaFrankie	Rhizome	Traumatic injury	Treat lung ailment, rheumatism, menstrual disturbance, mammitis, cuts, bruises, kidney diseases, and also to activate blood circulation and to alleviate pain	Xu et al. (2021)
*Tanacetum tatsienense* var. *tanacetopsis* (W. W. Smith) Grierson	Whole plant	Rheumatism; Dyspepsia	Relieve pain, blood bleeding	Li et al. (2009)
*Apios carnea* (Wall.) Benth.	Root	Cough	Deficiency of the kidney, lumbago	Lu (1988)
*Stauntonia angustifolia* (Wall.) R.Br. ex Wall.	Stem	Traumatic injury	Stomach pain, rheumatism pain	Zhu (1991)
*Fragaria vesca* L.	Root	Cold	Tuberculosis, pus and blood in the chest	Luo (1997)
*Chaenomeles lagenaria* (Loisel.) Koidz.	Fruit	Dysentery	Traumatic injury, bones and muscles pain	Zhang et al. (2014)
*Persicaria capitata* (Buch.-Ham. ex D. Don) H. Gross	Whole plant	Nephritis	Urinary infection	Liu et al. (2021)
*Tremella aurantialba* Bandoni et Zang	Fruit body	Tumour	Tuberculosis, asthenia cough, cold, phlegm, asthma, hypertension	Luo (1997)
*Hypocrella bambusae* (Berk. et Br.) Sacc.	Fruit body	Amygdalitis	Rheumatoid arthritis, stomach problems and fungal infections	Chen et al. (2019)
*Thamnolia vermicularia* (Ach.) Asahina	Whole plant	Asthma	Treat sore throats, hypertension, cough caused by lung-heat, fever and neurasthenia	Xiang et al. (2013)
*Impatiens arguta* Hook.f. & Thomson	Whole plant	Skin itch	Stomachache	Zhu (1991)
*Sonchus oleraceus* (L.) L.	Whole plant	Animal bite	Enteritis, dysentery, acute icteric hepatitis, appendicitis, mastitis, stomatitis, pharyngitis, tonsillitis, tuberculosis	Zhou and Zheng. (2010)
*Cinnamomum glanduliferum* (Wall.) Meisn.	Leaf	Skin allergy; Skin itch	Cold, heat stroke, bronchitis, food stagnation, gas distension, stomachache, diarrhea, rheumatic arthralgia	Zhou and Zheng. (2010)
*Alnus nepalensis* D.Don	Bark	Wound	Dysentery, diarrhea	Long et al. (1999)

## 4 Discussion

### 4.1 Medicinal Dietary Plants

In long-term production and living practice, the Dulong use the surrounding wild edible plants to provide food and to alleviate food shortages. There is much overlap between medicine and food, and plants can simultaneously be used as food and medicine (Zhang et al., 2015). Many plants in local food cultures are inseparable from traditional therapeutic systems. Based on our previous research on wild edible plants of the Dulong, we found that among the medicinal plants used by the Dulong, 62 species are edible (Supplementary Table S1). *Angiopteris esculenta*, a fern species endemic to northwest Yunnan, can weigh more than 20 kg per plant. The rhizome of *A. esculenta* can be crushed and applied externally to treat skin pruritus (Lu et al., 2021; Cheng et al., 2022). In the past, *Cardiocrinum giganteum* was an important food source plant for the Dulong. The bulb of *C. giganteum* can be directly processed into starch after washing and can also be used to make wine. The starch of *C. giganteum* can be used to treat indigestion. *Caryota obtusa* is a species of high cultural importance in the daily life of the Dulong. *C. obtusa* has multiple values, including edible, material, medicinal, and ornamental uses. The stem of *C. obtusa* is rich in starch, which can be processed into sago used to make *baba* (local cake), and the processed sago can be used for dysentery and dyspepsia. Stem tips and unflowered bracts of young trees can also be used as vegetables. The trunk of *C. obtusa* is an excellent building material. *C. obtusa* is a good ornamental plant with a beautiful tree shape and neat leaves. These medicinal dietary plants play an important role in ensuring food and medical safety. In the future, medicinal dietary plants with economic potential can be developed to make them a source of income for residents and promote local economic development.

The Dulong people are considered to be the last hunting group and seasonal foragers in China (Song, 1999; Fortier, 2014; Hitchcock, 2021). Collecting and hunting were important means for the Dulong people to maintain their daily lives in the past (Du and Chen, 2019). In the long process of interaction with the living environment, many medicinal plants and wild edible plants were consumed, and traditional ecological knowledge about them has been accumulated by local people because of the complicated topography, rainy weather, isolated and remote locality, poor transportation and abundant natural resources. Our previous study documented 148 species of wild edible plants used by the Dulong people (Cheng et al., 2022). Further studies were conducted on some wild edible plants (*Angiopteris esculenta*, *Caryota obtusa*, and *Maianthemum atropurpureum*), demonstrating that the local people’s practice of consuming wild edible plants is sustainable (Lu et al., 2021; Xu et al., 2021). In this study, we found that the Dulong people had high consistency in the treatment of diseases of the digestive system, injury, and musculoskeletal system, which may be related to its frequent gathering and hunting activities in the past. The Dulongjiang valley is one of the richest areas of wildlife in China. It is the core area of the Gaoligongshan National Nature Reserve, which is known as the “Animal and Plant Gene Bank”. Hunting has been banned in order to conserve wildlife. Fishing is also prohibited in some seasons.

### 4.2 Threats to Traditional Knowledge Associated With Medicinal Plants

**FIGURE 3:**
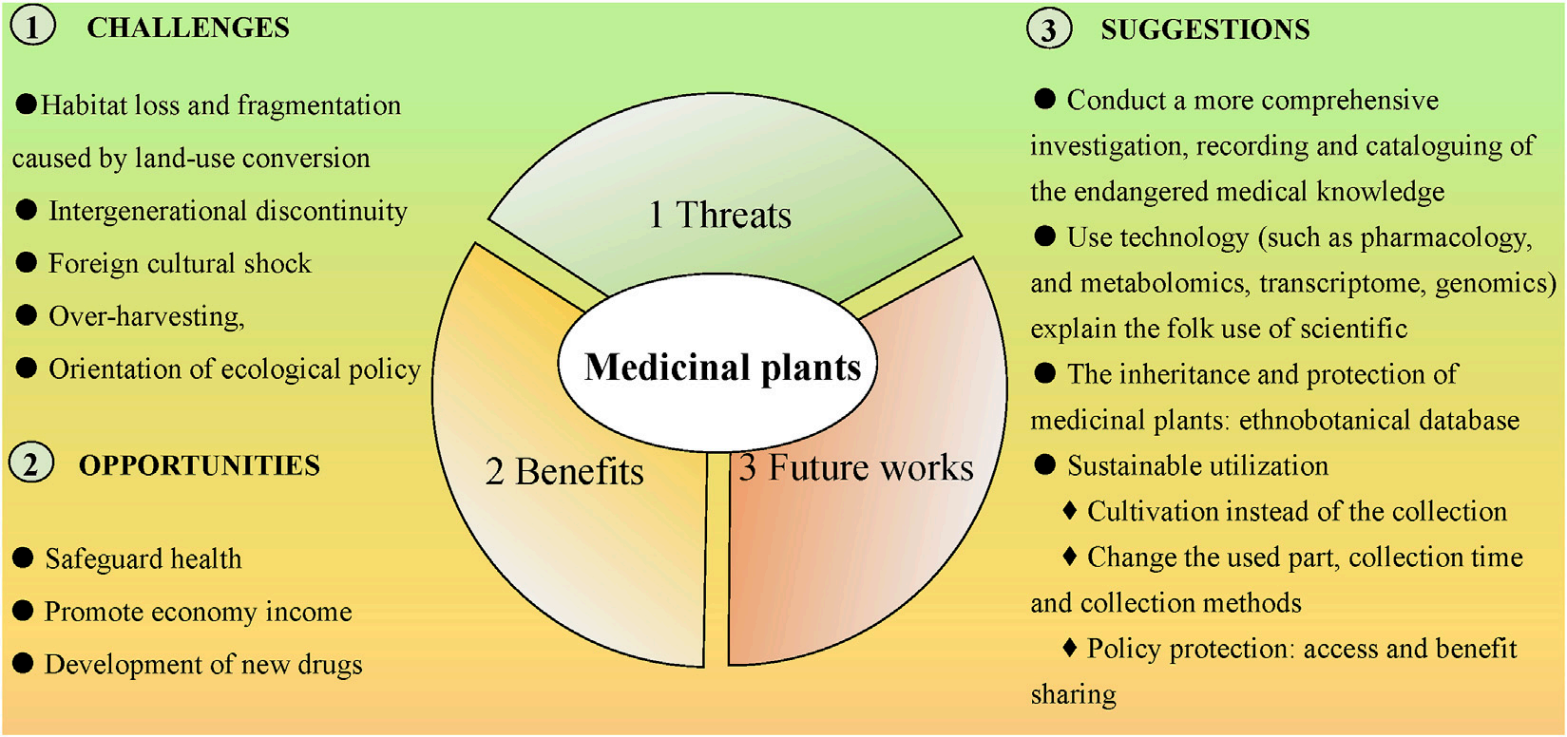
Threats, benefits, and future works of medicinal plants.

Medicinal plants and related traditional knowledge are the basis of the healthy and sustainable development of traditional medicine and provide a guarantee for the survival and health of human beings. The investigation of medicinal plants and related traditional knowledge is of great significance for the conservation of biodiversity, sustainable utilization of resources, and breeding of new varieties (Zhang et al., 2018). With the rapid development of the economy, science, and technology, the improvement of people’s living conditions, and the transformation of lifestyle, more and more people begin to pay attention to traditional medicine, especially in the aspects of health preservation and medicated diet. More and more traditional herbal medicine has been demystified by modern approaches, and the scientific nature of their treatment has been widely recognized (Luo et al., 2020). However, traditional medicine still faces many challenges, which may cause some small populations to lose traditional knowledge of medicinal plants. The major threat to medicinal plants is habitat loss and fragmentation caused by land-use conversion, intergenerational discontinuity, mainstream cultural shock, over-harvesting, and orientation of ecological policy (Figure 3).

#### 4.2.1 Imported alien species and cultural shock

The import of mainstream culture has made a great impact on its traditional knowledge. Many varieties of herbicides and pesticides began to flow into the Dulongjiang region. Besides, a large number of exotic ornamental plants are planted along the banks of the Dulongjiang region, and fewer native ornamental plants are used. The application of these herbicides and the spread of alien species will cause damage to the local medicinal plants. It will also be detrimental to biodiversity conservation in the Dulongjiang region.

#### 4.2.2 Intergenerational discontinuity

The knowledge of traditional medicine is mostly in the hands of the elderly. Most of the younger generations of the Dulong are not very interested in this knowledge. As the elderly gradually pass away, the traditional knowledge is in danger of disappearing.

#### 4.2.3 Habitat loss and fragmentation caused by land-use conversion

Monoculture is the major cause. The Dulong people have planted large number of *Amomum tsaoko*. Although it’s an important economic plant, single-crop plantation will reduce the diversity of understory plants, which is not conducive to the survival of other animals and plants.

#### 4.2.4 Orientation of ecological policy

The Dulongjiang region is located in the core area of the Gaoligongshan National Nature Reserve. As a forest border community, the Dulong inevitably have a high degree of dependence on natural resources. In recent years, nature reserves have implemented a series of protection measures, such as natural protection forest projects, relocation, returning farmland to forests, etc. The purpose is to gradually reduce this dependence, but sometimes the opposite effect emerged. Previous studies on the livelihood of the Dulong indicated that the implementation of the environmental protection project in the Dulongjiang region has had a huge impact on the local area, including changes in the traditional livelihood and lifestyle of the local Dulong, reduction in arable land, reduced food production, and reduced income (Li, 2008). These problems may be the reason why forest marginal communities rely too much on medicinal plants. How to properly handle the contradiction between forest marginal community economic development, rational use of forest resources, and animal and plant protection is a long-term task in the future.

### 4.3 The Future Work of Medicinal Plants Associated With Traditional Knowledge

Traditional medicinal knowledge has potential contributions to drug development and safeguarding people’s health (Pei and Huai, 2007). Traditional medical knowledge is disappearing faster and faster, especially for the medicine of ethnic minorities in remote areas, which is more obvious.

In the future, four aspects of work for traditional medicine should be carried out. 1) The first step is to conduct a more comprehensive investigation, recording and cataloging of the endangered medicinal knowledge of Dulong through ethnobotanical methods; 2) Followed by research with modern ethnobotanical methods to provide guidance, using new technology (such as metabolomics, transcriptomics, or genomics) to explain the folk use of medicines with scientific evidence. Fully revealing the scientific basis of traditional medicine is conducive to traditional knowledge protection and sustainable development; 3) The continuity and conservation of medicinal plants: Establish a comprehensive Dulong ethnobotanical database, input the utilization information of relevant medicinal plant and upload pictures of plants. It is not only conducive to the dissemination and identification of traditional knowledge but also provides information and reference for the long-term preservation and follow-up research of traditional knowledge (Figure 3).

To make sure of the sustainable use of these plants, there are several suggestions as follows: With the support of science and technology, the cultivation of medicinal plants should be promoted instead of the collection of wild medicinal plants. Innovative use of medicinal plants can be proposed for their conservation and sustainable uses, such as the use of parts, collection times and collection methods. Policies can be issued to promote access and benefit-sharing system of the local traditional medical knowledge. More specifically, if the traditional knowledge is utilized, the benefits should be shared. Thus, it is necessary to make the traditional knowledge holders, providers, and users establish a fair and benefit-sharing relationship and safeguard the interests of local people, which can also be conducive to the protection of the traditional medical knowledge.

## 5 Conclusion

This study is the first medicinal ethnobotanical survey in Dulong area. A total of 105 medicinal plant species used by Dulong people were investigated and recorded, reflecting the Dulong have rich traditional knowledge about medicinal plants, which plays an important role in their healthcare. *Coptis teeta*, *Acorus calamus*, *Ophiocordyceps sinensis*, *Tanacetum tatsienense* var. *tanacetopsis*, and *Paris polyphylla* var. *yunnanensis* had high RFC. Dulong people had high consistency in the treatment of diseases in the digestive system, injury, and musculoskeletal system. In this study, we reported four species which have not been previously recorded as medicines and reported 19 new therapeutic uses of 17 medicinal species. Further studies on their chemical composition and pharmacological activity are needed. Nowadays, traditional knowledge has seriously been threatened due to recent human activities and environmental degradation. In the future, new approaches will be used to demystify traditional medicine. Measures are urgently needed to promote the inheritance of the traditional knowledge. Also, the sustainable use of medicinal plants needs to be ensured to improve the economic development of local people based on the premise of biodiversity conservation.

## Data Availability

The original contributions presented in the study are included in the article/Supplementary Material, further inquiries can be directed to the corresponding author.

## References

[B1] Al-Fatimi M. (2019). Ethnobotanical Survey of Medicinal Plants in central Abyan Governorate, Yemen. J. Ethnopharmacol. 241, 111973. 10.1016/j.jep.2019.111973 31146001

[B2] Ali S. I. Sheikh W. M. Rather M. A. Venkatesalu V. Muzamil Bashir S. Nabi S. U. (2021). Medicinal Plants: Treasure for Antiviral Drug Discovery. Phytother. Res. 35, 3447–3483. 10.1002/ptr.7039 33590931 PMC8013762

[B3] Bailly C. (2021). The Traditional and Modern Uses of Selaginella Tamariscina (P.Beauv.) Spring, in Medicine and Cosmetic: Applications and Bioactive Ingredients. J. Ethnopharmacol. 280, 114444. 10.1016/j.jep.2021.114444 34302944

[B4] Barkaoui M. Katiri A. Boubaker H. Msanda F. (2017). Ethnobotanical Survey of Medicinal Plants Used in the Traditional Treatment of Diabetes in Chtouka Ait Baha and Tiznit (Western Anti-atlas), Morocco. J. Ethnopharmacol. 198, 338–350. 10.1016/j.jep.2017.01.023 28109915

[B5] Chen Y. G. Liu Y. C. Guo Kai. Li S. H. Niu X. M. (2019). Chemical Constituents of *Shiraia bambusicola* and *Hypocrella Bambusae* and Their Cytotoxic Activity. Nat. Prod. Res. Dev. 31 (06), 1006–1011. 10.16333/j.1001-6880.2019.6.013

[B6] Cheng Z. Lu X. Lin F. Naeem A. Long C. (2022). Ethnobotanical Study on Wild Edible Plants Used by Dulong People in Northwestern Yunnan, China. J. Ethnobiol. Ethnomed. 18, 3. 10.1186/s13002-022-00501-3 35062974 PMC8781162

[B7] Cheng Z. Luo B. Fang Q. Long C. (2020a). Ethnobotanical Study on Plants Used for Traditional Beekeeping by Dulong People in Yunnan, China. J. Ethnobiol. Ethnomed. 16 (1), 61. 10.1186/s13002-020-00414-z 33054863 PMC7559768

[B8] Cheng Z. Shu H. Zhang S. Luo B. Gu R. Zhang R. (2020b). From Folk Taxonomy to Species Confirmation of *Acorus* (Acoraceae): Evidences Based on Phylogenetic and Metabolomic Analyses. Front. Plant Sci. 11, 965. 10.3389/fpls.2020.00965 32670345 PMC7327505

[B9] Chi C. Zhang Q. Z. Yu C. X. (1991). Study on *Viburnum Cylindricum* of Dai Medicine. J. Yunnan Univ. Tradit. Chin. Med. 14 (03), 39–40. 10.19288/j.cnki.issn.1000-2723.1991.03.013

[B10] Cox P. A. (2000). Will Tribal Knowledge Survive the Millennium? Science 287 (5450), 44–45. 10.1126/science.287.5450.44 10644221

[B11] Dubasque D. Chen Q. D. (2019). Les réflexions, productions et recommandations du groupe de travail "?Numérique et travail social?» du Haut Conseil du travail social. J. Guangxi Univ. Natl. (Philosophy Soc. Sci. Edition) 28 (4), 89–98. 10.3917/vsoc.194.0089

[B12] Dwi A. (2009). Traditionally Utilization of *Selaginella*; Field Research and Literature Review. Nusantara Biosci. 1 (3), 53–54.

[B13] Fortier J. (2014). “Regional hunter-gatherer Traditions in South-East Asia,” in Oxford Handbook of the Archaeology and Anthropology of hunter-gatherers. Editors Cummings V. Jordan P. Zvelebil M. (Oxford: Oxford University Press), 1010–1030.

[B14] Geng Y. Hu G. Ranjitkar S. Wang Y. Bu D. Pei S. (2017). Prioritizing Fodder Species Based on Traditional Knowledge: a Case Study of Mithun (*Bos frontalis*) in Dulongjiang Area, Yunnan Province, Southwest China. J. Ethnobiol. Ethnomed. 13 (1), 24. 10.1186/s13002-017-0153-z 28472968 PMC5418811

[B15] Geng Y. Ranjitkar S. Yan Q. He Z. Su B. Gao S. (2020). Nutrient Value of Wild Fodder Species and the Implications for Improving the Diet of Mithun (*Bos frontalis*) in Dulongjiang Area, Yunnan Province, China. Plant Divers. 42 (6), 455–463. 10.1016/j.pld.2020.09.007 33733013 PMC7936111

[B16] Gras A. Parada M. Vallès J. Garnatje T. (2021). The Role of Traditional Plant Knowledge in the Fight against Infectious Diseases: A Meta-Analytic Study in the Catalan Linguistic Area. Front. Pharmacol. 12, 744616. 10.3389/fphar.2021.744616 34707501 PMC8543157

[B17] Hitchcock R. K. (2021). Characteristics of hunter-gatherers in Asia. Senri Ethnol. Stud. 106, 253–273.

[B18] Hong W. Zhang Y. Yang J. Xia M. Y. Luo J. F. Li X. N. (2019). Alkaloids from the Branches and Leaves of *Elaeocarpus angustifolius* . J. Nat. Prod. 82 (12), 3221–3226. 10.1021/acs.jnatprod.8b01027 31736307

[B19] Huang J. Long C. (2007). *Coptis Teeta*-based Agroforestry System and its Conservation Potential: a Case Study from Northwest Yunnan. Ambio 36 (4), 343–349. 10.1579/0044-7447(2007)36[343:ctasai]2.0.co;2 17626473

[B20] Huang J. Long C. L. (2006). Traditional Cultivation of *Coptis Teeta* and its Values in Biodiversity Conservation. Biodiversity Sci. 14 (1), 79–86. 10.1360/biodiv.050092

[B21] Huang S. S. Huang C. H. Ko C. Y. Chen T. Y. Cheng Y. C. Chao J. (2021). An Ethnobotanical Study of Medicinal Plants in Kinmen. Front. Pharmacol. 12, 681190. 10.3389/fphar.2021.681190 35222004 PMC8864234

[B22] International Society of Ethnobiology (2006). International Society of Ethnobiology Code of Ethics (With 2008 Additions). Available at: http://ethnobiology.net/code-of-ethics/.

[B23] IUCN (2021). The IUCN Red List of Threatened Species. Version 2021-1. Available at: https://www.iucnredlist.org.

[B24] Jurikova T. Skrovankova S. Mlcek J. Balla S. Snopek L. (2018). Bioactive Compounds, Antioxidant Activity, and Biological Effects of European cranberry (*Vaccinium Oxycoccos*). Molecules 24 (1). 10.3390/molecules24010024 PMC6337168 30577610

[B25] Kang D. G. Yin M. H. Oh H. Lee D. H. Lee H. S. (2004). Vasorelaxation by Amentoflavone Isolated from *Selaginella Tamariscina* . Planta Med. 70 (08), 718–722. 10.1055/s-2004-827201 15326548

[B26] Katavic P. L. Venables D. A. Rali T. Carroll A. R. (2007). Habbemines A and B, Pyrrolidine Alkaloids with Human delta-opioid Receptor Binding Affinity from the Leaves of Elaeocarpus Habbemensis. J. Nat. Prod. 70 (5), 866–868. 10.1021/np060577f 17388627

[B27] Li F. S. Weng J. K. (2017). Demystifying Traditional Herbal Medicine with Modern Approach. Nat. Plants 3 (8), 17109. 10.1038/nplants.2017.109 28758992

[B28] Li H. (1994). Delineation and Feature of Dulongjiang Region flora. Plant Divers. S6, 1–100.

[B29] Li J. M. (2008). Study on Ecological protection and National Livelihood Sustainable Development-Taking Dulong Nationality in Dulongjiang Region as an Example. Social Sci. Yunnan 2 (03), 81–85.

[B30] Li J. M. (2012). Study on the Traditional Knowledge of the Utilization of the Dulong Wild Plants. Academic Exploration, 63–68.04

[B31] Li Q. F. Ouyang J. Yang Y. Wen M. L. (2009). Study on Essential Oil of *Cancrinia Discoidea* . J. Yunnan Univ. Tradit. Chin. Med. 32 (02), 20–22. 10.19288/j.cnki.issn.1000-2723.2009.02.008

[B32] Li R. Dao Z. Li H. (2011). Seed Plant Species Diversity and Conservation in the Northern Gaoligong Mountains in Western Yunnan, China. Mountain Res. Development 31 (2), 160–165. 10.1659/MRD-JOURNAL-D-10-00056.1

[B33] Lin F. Luo B. Long B. Long C. (2019). Plant Leaves for Wrapping Zongzi in China: an Ethnobotanical Study. J. Ethnobiol. Ethnomed. 15, 63. 10.1186/s13002-019-0339-7 31829257 PMC6907129

[B34] Liu B. Guo Z. Y. Bussmann R. Li F. F. Li J. Q. Hong L. Y. (2016). Ethnobotanical Approaches of Traditional Medicine Studies in Southwest China: A Literature Review. J. Ethnopharmacol. 186, 343–350. 10.1016/j.jep.2016.02.040 26997553

[B35] Liu Y. L. Sui Y. Hu C. X. Liu M. Zou M. Xu J. S. (2021). Summary of the Pharmacological Activities of Miao Medicine *Polygonum* . J. Guizhou Univ. Tradit. Chin. Med. 43 (01), 81–84. 10.16588/j.cnki.issn2096-8426.2021.01.021

[B36] Long C. L. Li H. Zhou Y. L. Dao Z. L. Abe T. (1999). Ethnobotanical Studies in Gaoligong Mountains: The Dulong Ethnic Group. Plant Divers. S1, 137–144.

[B37] Lu K. H. (1988). Traditional Chinese Medicine of Miao. Guizhou People's Publishing House.

[B38] Lu X. P. Cheng Z. Long C. L. (2021). *Angiopteris Esculenta*, a Traditional Edible Plant Consumed by Dulong People. Guihaia, 1–17. 10.11931guihaia/gxzw202106024

[B39] Luo B. Liu Y. Liu B. Liu S. Zhang B. Zhang L. (20182018). Yao Herbal Medicinal Market during the Dragon Boat Festival in Jianghua County, China. J. Ethnobiol. Ethnomed. 14 (1), 61. 10.1186/s13002-018-0260-5 PMC6192344 30333030

[B40] Luo D. S. (1997). Traditional Chinese Medicine of Tibetan. Nationalities Publishing House.

[B41] Luo L. Jiang J. Wang C. Fitzgerald M. Hu W. Zhou Y. (2020). Analysis on Herbal Medicines Utilized for Treatment of COVID-19. Acta Pharm. Sin. B 10 (7), 1192–1204. 10.1016/j.apsb.2020.05.007 32834949 PMC7251357

[B42] Mi X. C. Feng G. Hu Y. B. Zhang J. Chen L. Corlett R. T. (2021). The Global Significance of Biodiversity Science in China: an Overview. Natl. Sci. Rev. 8. nwab032. 10.1093/nsr/nwab032/6147049 34694304 PMC8310773

[B43] Miara M. D. Bendif H. Ait Hammou M. Teixidor-Toneu I. (2018). Ethnobotanical Survey of Medicinal Plants Used by Nomadic Peoples in the Algerian Steppe. J. Ethnopharmacol. 219, 248–256. 10.1016/j.jep.2018.03.011 29548971

[B44] Ojha S. N. Tiwari D. Anand A. Sundriyal R. C. (2020). Ethnomedicinal Knowledge of a Marginal hill Community of Central Himalaya: Diversity, Usage Pattern, and Conservation Concerns. J. Ethnobiol. Ethnomed. 16, 29. 10.1186/s13002-020-00381-5 32448334 PMC7245762

[B45] Ozukum A. Changkija S. Tripathi S. K. (2019). Ethnobotanical Studies on the Khiamniungan Tribe in Tuensang District of Nagaland, Northeast India: Ethnomedicinal Plants. Pleione 13 (1), 070. 10.26679/Pleione.13.1.2019.070-081

[B46] Pardo-de-Santayana M. Macía M. J. (2015). Biodiversity: The Benefits of Traditional Knowledge. Nature 518 (7540), 487–488. 10.1038/518487a 25719661

[B47] Pei S. J. Huai H. Y. (2007). Ethnobotany. Shanghai Science and Technology Press.

[B48] Prasannan P. Jeyaram Y. Pandian A. Raju R. Sekar S. (2020). A Review on Taxonomy, Phytochemistry, Pharmacology, Threats and Conservation of *Elaeocarpus* L. (Elaeocarpaceae). Bot. Rev. 86, 298–328. 10.1007/s12229-020-09229-9

[B49] Quave C. L. Pieroni A. (2015). A Reservoir of Ethnobotanical Knowledge Informs Resilient Food Security and Health Strategies in the Balkans. Nat. Plants 1 (2), 14021. 10.1038/nplants.2014.21 27246758

[B50] Rahman I. U. Afzal A. Iqbal Z. Ijaz F. Ali N. Shah M. (2018). Historical Perspectives of Ethnobotany. Clin. Dermatol. 37, 382–388. 10.1016/j.clindermatol.2018.03.018 31345328

[B51] Samy R. P. Gopalakrishnakone P. (2007). Current Status of Herbal and Their Future Perspectives. Nat. Prec 1176. 10.1038/npre.2007.1176.1

[B52] Schmidt B. M. Cheng D. M. K. (2017). Ethnobotany (A Phytochemical Perspective). John Wiley & Sons.

[B53] Sheng-Ji P. (2001). Ethnobotanical Approaches of Traditional Medicine Studies: Some Experiences from Asia. Pharm. Biol. 39 (sup1), 74–79. 10.1076/phbi.39.s1.74.0005 21554174

[B54] Shu H. Zhang S. Lei Q. Zhou J. Ji Y. Luo B. (2018). Ethnobotany of *Acorus* in China. Acta Soc. Bot. Pol. 87 (2), 3585. 10.5586/asbp.3585

[B55] Song E. (1999). “The Dulong,” in The Cambridge Encyclopedia of hunters and Gatherers. Editors Lee R. B. Daly R. H. Daly R. (Camabridge: Cambridge University Press), 303–306.

[B56] Staub P. O. Geck M. S. Weckerle C. S. Casu L. Leonti M. (2015). Classifying Diseases and Remedies in Ethnomedicine and Ethnopharmacology. J. Ethnopharmacol. 174, 514–519. 10.1016/j.jep.2015.08.051 26342522

[B57] Sun J. Dao Z. Li R. (2011). Investigation on Common Plant Medicine in Dulong Folk in Yunnan. Chin. J. Ethnomedicine Ethnopharmacy 20 (19), 6–8.

[B58] Tardío J. Pardo-de-Santayana M. (2008). Cultural Importance Indices: A Comparative Analysis Based on the Useful Wild Plants of Southern Cantabria (Northern Spain)1. Econ. Bot. 62, 24–39. 10.1007/s12231-007-9004-5

[B59] Trotter R. Logan M. (1986). “Informant Consensus, a New Approach for Identifying Potentially Effective Medicinal Plants,” in Plants in Indigenous Medicine and Diet, Biobehavioural Approaches. Editor Etkin N. L. (Bedford Hills, NY: Redgrave Publishers), 91–112.

[B60] Xiang W. J. Wang Q. Q. Ma L. Hu L. H. (2013). β-Orcinol-type Depsides from the Lichen Thamnolia Vermicularis. Nat. Prod. Res. 27 (9), 804–808. 10.1080/14786419.2012.704374 22799538

[B61] Xu L. Wang Y. Ji Y. Li P. Cao W. Wu S. (2021). Nutraceutical Study on *Maianthemum Atropurpureum*, a Wild Medicinal Food Plant in Northwest Yunnan, China. Front. Pharmacol. 12, 710487. 10.3389/fphar.2021.710487 34393791 PMC8363226

[B62] Yang J. Li Z. J. Song N. L. (2016). Study on Chemical Compositions of *Viburnum Cylindricum* of Dai Medicine. Yunnan J. Tradit. Chin. Med. Materia Med. 37 (10), 72–74. 10.16254/j.cnki.53-1120/r.2016.10.034

[B63] Yang Y. J. Xie S. Q. Yang S. C. Xu X. Z. Liang Y. L. (2011). The Progress on *Coptis Teeta* Wall. J. Anhui Agric. Sci. 39 (23), 14037–14038. 10.13989/j.cnki.0517-6611.2011.23.178

[B64] Yao R. Heinrich M. Wei J. Xiao P. (2021). Cross-Cultural Ethnobotanical Assembly as a New Tool for Understanding Medicinal and Culinary Values-The Genus Lycium as A Case Study. Front. Pharmacol. 12. 10.3389/fphar.2021.708518 PMC8322658 34335270

[B65] Zhang H. F. Yang X. H. (2012). Asian Medicine: Protect Rare Plants. Nature 482, 35. 10.1038/482035e 22297962

[B66] Zhang L. Zhang Y. Pei S. Geng Y. Wang C. Yuhua W. (2015). Ethnobotanical Survey of Medicinal Dietary Plants Used by the Naxi People in Lijiang Area, Northwest Yunnan, China. J. Ethnobiol. Ethnomed. 11, 40. 10.1186/s13002-015-0030-6 25962397 PMC4449607

[B67] Zhang L. Zhuang H. Zhang Y. Wang L. Zhang Y. Geng Y. (2018). Plants for Health: an Ethnobotanical 25-year Repeat Survey of Traditional Medicine Sold in a Major Marketplace in North-west Yunnan, China. J. Ethnopharmacol. 224, 119–125. 10.1016/j.jep.2018.05.029 29800743

[B68] Zhang S. Y. Han L. Y. Zhang H. Xin H. L. (2014). *Chaenomeles Speciosa*: A Review of Chemistry and Pharmacology. Biomed. Rep. 2, 12–18. 10.3892/br.2013.193 24649061 PMC3917013

[B69] Zhou G. Y. Wu S. Y. Hu Z. R. Li K. M. Tang C. F. Zhang L. Q. (2011). Survey of Agricultural Biological Resources and Their Traditional Knowledge in Dulong Community. J. Plant Genet. Resour. 12 (6), 998–1003. 10.13430/j.cnki.jpgr.2011.06.001

[B70] Zhou Y. C. Zheng J. (2010). Traditional Medicine of Nujiang Region. Yunnan Science and Technology Press.

[B71] Zhu Z. Y. (1991). Traditional Chinese Medicine Resources of Dali. Yunnan Nationalities Publishing House.

