# Supplementary material for: Medicinal Plants and Fungi Traditionally Used by Dulong People in Northwest Yunnan, China
Source: Front Pharmacol. 2022 May 9;13:895129. doi: 10.3389/fphar.2022.895129 (PMC9124798; doi:10.3389/fphar.2022.895129)
Supplement: Supplementary file 1 [file Table1.docx]

Supplementary Material

**Supplementary Table 1** | The inventory of medicinal plants and fungi used by Dulong people

| **Scientific name** | **Family name** | **Local name** | **Medicinal parts** | **Processing method** | **Medicinal effect** | **Protection level** | **Edible** | **Relative frequency of citation** | **Specimen number** |
| --- | --- | --- | --- | --- | --- | --- | --- | --- | --- |
| **Angiosperm** |  |  |  |  |  |  |  |  |  |
| *Acorus calamus* L. | Acoraceae | zen bu re | Rhizome | Processed into powder, decoction/oral | Gastritis; Animal bite | LC | Y | 0.12 | DLZ0044 |
| *Saurauia napaulensis* DC. | Actinidiaceae | da bu qiu | Root | Crushed/external application | Traumatic injury | LC | Y | 0.02 | DLZ0046 |
| *Viburnum cylindricum* Buch.-Ham. ex D. Don | Adoxaceae | ri bu | Leaf | Decoction or alcohol maceration/oral | Cold |  |  | 0.02 | DLZ0123 |
| *Allium hookeri* Thwaites | Amaryllidaceae | su, be ceng | Whole plant | Cooked | Traumatic injury |  | Y | 0.01 | DLZ0021 |
| *Toxicodendron vernicifluum* (Stokes) F.A. Barkley | Anacardiaceae | de ki | Seed | Processed into oil, stewed with chicken | Postpartum recovery | LC | Y | 0.03 | DLZ0172 |
| *Angelica dahurica* (Hoffm.) Benth. & Hook.f. ex Franch. & Sav. | Apiaceae | da bu cui | Root | Decoction/oral | Rheumatism |  | Y | 0.03 | DLZ0041 |
| *Heracleum hemsleyanum* Diels | Apiaceae | dong a | Root | Decoction/oral | Rheumatism |  | Y | 0.03 | DLZ0020 |
| *Foeniculum vulgare* Mill. | Apiaceae | —— | Whole plant | Cooked | Stomach ache; Neurasthenia; Tinnitus | LC | Y | 0.02 | DLZ0162 |
| *Colocasia esculenta* (L.) Schott | Araceae | gui | Tuber | Crushed/external application or smear with vinegar | Animal bite | LC | Y | 0.03 | DLZ0135 |
| *Arisaema erubescens* (Wall.) Schott | Araceae | ben ba ha | Tuber | Decoction/rinse | Traumatic injury; Animal bite |  |  | 0.02 | DLZ0120 |
| *Panax japonicus* (T.Nees) C.A.Mey. | Araliaceae | —— | Rhizome | Cooked | Bleeding |  | Y | 0.03 | —— |
| *Aralia elata* (Miq.) Seem. | Araliaceae | bang a | Root | Crushed/external application | Fracture | LC | Y | 0.01 | DLZ0166 |
| *Caryota obtusa* Griff. | Arecaceae | a lei | Stem | Processed into powder, cooked | Dysentery; Dyspepsia | LC, (II) | Y | 0.06 | —— |
| *Polygonatum cirrhifolium* (Wall.) Royle | Asparagaceae | mu xia | Rhizome | Decoction or stewed with meat | Cough |  | Y | 0.06 | —— |
| *Maianthemum atropurpureum* (Franch.) LaFrankie | Asparagaceae | dong | Rhizome | Crushed/external application | Traumatic injury |  | Y | 0.04 | —— |
| *Tanacetum tatsienense* var. *tanacetopsis* (W. W. Smith) Grierson | Asteraceae | mu qiu | Whole plant | Decoction/oral | Rheumatism; Dyspepsia |  | Y | 0.08 | DLZ0100 |
| *Artemisia dubia* L. ex B.D.Jacks. | Asteraceae | si mer | Leaf | Crushed/external application | Wound |  |  | 0.07 | DLZ0011 |
| *Pseudognaphalium* affine (D.Don) Anderb. | Asteraceae | di ka rang xin, wen | Whole plant | Decoction/oral | Cold; Asthma; Bronchitis |  | Y | 0.02 | DLZ0016 |
| *Sonchus oleraceus* (L.) L. | Asteraceae | da nu | Whole plant | Crushed/external application | Animal bite |  | Y | 0.02 | DLZ0174 |
| *Saussurea obvallata* (DC.) Edgew. | Asteraceae | —— | Whole plant | Decoction/oral | Rheumatic arthritis; Irregular menstruation; Heart disease |  | Y | 0.06 | —— |
| *Taraxacum mongolicum* Hand.-Mazz. | Asteraceae | ba ko er jia | Whole plant | Decoction/oral | Cold |  | Y | 0.02 | DLZ0109 |
| *Nabalus tatarinowii* (Maxim.) Nakai | Asteraceae | —— | Latex | Crushed/external application | Wound |  |  | 0.02 | DLZ0110 |
| *Senecio scandens* Buch.-Ham. ex D.Don | Asteraceae | rong me ra | Whole plant | Crushed/external application | Skin itch | LC |  | 0.01 | DLZ0108 |
| *Impatiens arguta* Hook.f. & Thomson | Balsaminaceae | ze li | Whole plant | Crushed/external application | Skin itch |  |  | 0.02 | DLZ0071 |
| *Begonia acetosella* Craib | Begoniaceae | xie jiang leng | Stem、leaf | Crushed/Chew or cooked with fish | Anemia |  | Y | 0.03 | DLZ0084 |
| *Berberis pruinosa* Franch. | Berberidaceae | a qiu xi sha | Root | Decoction/oral | Cold |  |  | 0.02 | DLZ0167 |
| *Mahonia fortunei* (Lindl.) Fedde | Berberidaceae | —— | Root、leaf | Decoction/oral | Cough; Headache; Tinnitus |  |  | 0.02 | DLZ0113 |
| *Alnus nepalensis* D.Don | Betulaceae | s me | Bark | Decoction/oral or crushed/external application | Wound; Dysentery; Diarrhea | LC |  | 0.02 | —— |
| *Codonopsis* spp. | Campanulaceae | —— | Root | Decoction/oral | Deficiency of the kidney |  | Y | 0.04 | —— |
| *Lobelia clavata* E.Wimm. | Campanulaceae | li gen bu la | Whole plant | Crushed/external application | Rheumatism |  |  | 0.02 | DLZ0168 |
| *Dipsacus asper* Wall. ex C.B. Clarke | Caprifoliaceae | mu rang mu long | Rhizome | Crushed/external application | Fracture; Rheumatism |  |  | 0.02 | DLZ0103 |
| *Silene baccifera* (L.) Roth | Caryophyllaceae | ge lang la bu | Whole plant | Crushed/external application | Fracture; Rheumatic arthritis |  |  | 0.02 | DLZ0049 |
| *Commelina communis* L. | Commelinaceae | da lai | Whole plant | Crushed/external application | Fracture |  |  | 0.02 | DLZ0030 |
| *Coriaria napalensis* Wall. | Coriariaceae | bi li | Root, leaf | Crushed/external application | Rheumatism |  |  | 0.01 | DLZ0121 |
| *Cornus capitata* Wall. | Cornaceae | de ji er | Bark, leaf | Decoction/oral | Hepatitis | LC | Y | 0.02 | DLZ0003 |
| *Thladiantha cordifolia* (Blume) Cogn. | Cucurbitaceae | dong gua | Root | Crushed/external application | Animal bite |  | Y | 0.01 | DLZ0165 |
| *Momordica subangulata* Blume | Cucurbitaceae | de ga qi gong | Seed | Fried | Intoxication; Gynaecopathia |  | Y | 0.01 | DLZ0045 |
| *Dioscorea pentaphylla* L. | Dioscoreaceae | e jing | Rhizome | Toasted | Dyspepsia |  | Y | 0.01 | DLZ0007 |
| *Dioscorea bulbifera* L. | Dioscoreaceae | ki | Tuber | Crushed/external application | Skin ulcer |  | Y | 0.01 | DLZ0169 |
| *Elaeocarpus lacunosus* Wall. ex Kurz | Elaeocarpaceae | me li | Fruit | Alcohol maceration/oral | Traumatic injury |  | Y | 0.01 | DLZ0151 |
| *Equisetum giganteum* L. | Equisetaceae | ji ben | Whole plant | Decoction/oral | Urinary infection | LC |  | 0.01 | DLZ0126 |
| *Vaccinium gaultheriifolium* (Griff.) Hook. f. ex C.B. Clarke | Ericaceae | ke rei | Fruit | Juice/chew | Rheumatism |  | Y | 0.01 | DLZ0002 |
| *Pueraria peduncularis* (Benth.) Benth. | Fabaceae | b ri | Rhizome | Toasted | Cough |  | Y | 0.06 | DLZ0006 |
| *Apios carnea* (Wall.) Benth. | Fabaceae | bu leng mu sa | Root | Decoction/oral | Cough |  |  | 0.01 | DLZ0036 |
| *Geranium sinense* R.Knuth | Geraniaceae | ge shan xiao | Whole plant | Decoction/oral | Stomachache |  |  | 0.01 | —— |
| *Juglans sigillata* Dode | Juglandaceae | bu | Leaf | Decoction/oral | Leucorrhea | LC | Y | 0.02 | DLZ0160 |
| *Juncus effusus* L. | Juncaceae | cu xin | Stem | Decoction/oral | Mouth sores; Urinary infectio | LC |  | 0.01 | DLZ0140 |
| *Perilla frutescens* (L.) Britton | Lamiaceae | si lang | Whole plant | Decoction/oral | Cold | LC | Y | 0.01 | DLZ0171 |
| *Isodon serra* (Maxim.) Kudô | Lamiaceae | —— | Whole plant | Decoction/oral | Hepatitis; Acute cholecystitis |  |  | 0.01 | —— |
| *Stauntonia angustifolia* (Wall.) R.Br. ex Wall. | Lardizabalaceae | go leng | Stem | Decoction/oral | Traumatic injury |  | Y | 0.01 | DLZ0033 |
| *Litsea pungens* Hemsl. | Lauraceae | na ba qiu | Fruit | Cooked | Dyspepsia | LC | Y | 0.03 | DLZ0042 |
| *Cinnamomum glanduliferum* (Wall.) Meisn. | Lauraceae | qiu | Leaf | Crushed/external application | Skin allergy; Skin itch | LC |  | 0.01 | DLZ0026 |
| *Litsea cubeba* (Lour.) Pers. | Lauraceae | de qiang | Fruit | Decoction/oral | Cold; Headache; Dyspepsia |  | Y | 0.01 | DLZ0037 |
| *Fritillaria cirrhosa* D.Don | Liliaceae | me ki | Bulb | Processed into powder, cooked | Diarrhea |  | Y | 0.05 | —— |
| *Cardiocrinum giganteum* (Wall.) Makino | Liliaceae | a bo | Bulb | Processed into powder, cooked | Indigestion |  | Y | 0.04 | DLZ0029 |
| *Lilium davidii* Duch. ex Elwes | Liliaceae | mu ji | Bulb | Processed into powder, cooked | Pertussis |  | Y | 0.02 | —— |
| *Magnolia rostrata* W.W.Sm. | Magnoliaceae | xiu cai | Bark | Decoction/oral | Stomachache | EN, (II) |  | 0.06 | DLZ0102 |
| *Urena lobata* L. | Malvaceae | —— | Whole plant | Crushed/external application | Animal bite | LC |  | 0.01 | DLZ0106 |
| *Paris polyphylla* var. *yunnanensis* (Franch.) Hand.-Mazz. | Melanthiaceae | bu a | Rhizome | Crushed/external application | Cut | VU, (II) |  | 0.08 | —— |
| *Stephania delavayi* Diels | Menispermaceae | ga lai mu | Tuber | Decoction/oral | Malaria; Rheumatism |  |  | 0.02 | —— |
| *Morus mongolica* (Bureau) C.K. Schneid. | Moraceae | sai ji | Bark | Decoction/oral | Cold; Cough | LC | Y | 0.01 | DLZ0152 |
| *Musa basjoo* Sieb. et Zucc. | Musaceae | ke long | Stem | Decoction/oral | Fever |  | Y | 0.01 | DLZ0145 |
| *Dendrobium nobile* Lindl. | Orchidaceae | —— | Stem | Crushed/external application | Fracture | (I) | Y | 0.08 | DLZ0031 |
| *Gastrodia elata* Blume | Orchidaceae | tian ma | Rhizome | Sliced and stewed with meat | Headache | VU, (II), II | Y | 0.07 | —— |
| *Bletilla striata* (Thunb.) Reichb.f. | Orchidaceae | gu lie | Rhizome | Processed into powder/oral with honey | Pneumonia | (II), II | Y | 0.05 | DLZ0104 |
| *Phytolacca acinosa* Roxb. | Phytolaccaceae | hong shen | Root | Decoction/oral | Rheumatism |  |  | 0.01 | DLZ0105 |
| *Plantago major* L. | Plantaginaceae | wa gui | Whole plant | Decoction/oral | Cold; Urinary infection; Gynecological | LC | Y | 0.07 | DLZ0001 |
| *Imperata cylindrica* (L.) Raeusch. | Poaceae | a ji | Rhizome | Decoction/oral | Jaundice | LC | Y | 0.02 | DLZ0111 |
| *Phyllostachys mannii* Gamble | Poaceae | gan | Leaf | Decoction/oral | Cold |  | Y | 0.02 | DLZ0043 |
| *Phragmites australis* (Cav.) Trin. ex Steud. | Poaceae | hao | Rhizome | Decoction/oral | Cough | LC |  | 0.01 | DLZ0150 |
| *Fagopyrum dibotrys* (Lehm.) Mansf. ex K.Hammer | Polygonaceae | bu lei、xie ri | Stem | Cooked | Amygdalitis | (II) | Y | 0.04 | DLZ0014 |
| *Persicaria nepalensis* (Meisn.) H.Gross | Polygonaceae | de beng/qi bu rai | Whole plant | Decoction/oral | Dysentery; Arthralgia |  |  | 0.01 | DLZ0068 |
| *Persicaria capitata* (Buch.-Ham. ex D.Don) H.Gross | Polygonaceae | qi bu rai | Whole plant | Decoction/oral | Urinary infection; Nephritis |  |  | 0.01 | DLZ0067 |
| *Rumex nepalensis* Spreng. | Polygonaceae | —— | Root, leaf | Decoction/oral | Phthisis; Hepatitis |  |  | 0.01 | DLZ0175 |
| *Semiaquilegia adoxoides* (DC.) Makino | Ranunculaceae | wu su | Tuber | Decoction/oral | Amygdalitis |  |  | 0.01 | DLZ0008 |
| *Coptis teeta* Wall. | Ranunculaceae | xiu za | Root | Decoction/oral | Diarrhea | EN, (II) |  | 0.15 | —— |
| *Aconitum ouvrardianum* Hand.-Mazz. | Ranunculaceae | bu la | Root | Crushed/external application | Rheumatism; Headache |  |  | 0.01 | —— |
| *Eriocapitella vitifolia* (Buch.-Ham. ex DC.) Nakai | Ranunculaceae | wa le bao xing | Root | Crushed/external application | Dyspepsia; Gastritis |  |  | 0.01 | DLZ0170 |
| *Agrimonia pilosa* Ledeb. | Rosaceae | —— | Whole plant | Decoction/oral | Inflammation | LC |  | 0.03 | DLZ0015 |
| *Fragaria vesca* L. | Rosaceae | xia jiu | Root | Decoction/oral | Cold | LC | Y | 0.01 | DLZ0009 |
| *Chaenomeles lagenaria* (Loisel.) Koidz. | Rosaceae | mo lang | Fruit | Decoction/wash | Dysentery |  | Y | 0.01 | DLZ0124 |
| *Potentilla discolor* Bunge | Rosaceae | ban xiao mo | Whole plant | Decoction/oral | Dyspepsia |  |  | 0.01 | —— |
| *Zanthoxylum capense* (Thunb.) Harv. | Rutaceae | a za pu | Fruit | Decoction/oral | Stomachache | LC | Y | 0.03 | DLZ0143 |
| *Houttuynia cordata* Thunb. | Saururaceae | wu su bu | Whole plant | Cooked | Cold |  | Y | 0.05 | DLZ0023 |
| *Schisandra* spp. | Schisandraceae | me long | Fruit | Alcohol maceration/oral | Diarrhea |  | Y | 0.03 | DLZ0039 |
| *Solanum nigrum* L. | Solanaceae | mu hen | Whole plant | Crushed/external application | Traumatic injury |  | Y | 0.01 | DLZ0130 |
| *Gonostegia triandra* (Blume) Miq. | Urticaceae | ji leng | Rhizome | Crushed/external application | Backache |  | Y | 0.02 | DLZ0013 |
|  |  |  |  |  |  |  |  |  |  |
| **Gymnosperm** |  |  |  |  |  |  |  |  |  |
| *Pinus yunnanensis* Franch. | Pinaceae | dang me | Turpentine | Crushed/external application | Traumatic injury | LC |  | 0.01 | DLZ0125 |
|  |  |  |  |  |  |  |  |  |  |
| **Fern** |  |  |  |  |  |  |  |  |  |
| *Cibotium barometz* (L.) J.Sm. | Cibotiaceae | de bu | Rhizome | Alcohol maceration/oral | Backache | (II), II | Y | 0.01 | —— |
| *Dicranopteris pedata* (Houtt.) Nakaike | Gleicheniaceae | jia ke | Rhizome | Crushed/external application | Urinary infection; Burn |  |  | 0.01 | DLZ0136 |
| *Phlegmariurus austrosinicus* (Ching) L.B. Zhang | Huperziaceae | da bu si | Leaf | Decoction/oral | Joint pain |  |  | 0.02 | DLZ0040 |
| *Angiopteris esculenta* Ching | Marattiaceae | da bu qie/mei leng | Rhizome | Crushed/external application | Skin pruritus |  | Y | 0.03 | DLZ0081 |
| *Lepisorus thunbergianus* (Kaulf.) Ching | Polypodiaceae | me de r | Rhizome | Decoction/oral | Pharyngalgia |  |  | 0.02 | DLZ0005 |
| *Selaginella uncinata* (Desv. ex Poir.) Spring | Selaginellaceae | long bu la | Whole plant | Decoction/oral | Jaundice |  |  | 0.01 | DLZ0048 |
| *Selaginella bisulcata* Spring | Selaginellaceae | me qing | Whole plant | Decoction/oral | Heart disease |  |  | 0.02 | DLZ0088 |
|  |  |  |  |  |  |  |  |  |  |
| **Fungi** |  |  |  |  |  |  |  |  |  |
| *Ophiocordyceps sinensis* (BerK.) Sacc. | Clavicipitaceae | chong cao | Whole plant | Alcohol maceration/oral | Deficiency of the kidney; Backache | VU | Y | 0.11 | —— |
| *Hericium erinaceus* (Bull. ex Fr.) Pers. | Hydnaceae | —— | Fruit body | Cooked or stewed with meat | Dyspepsia | LC | Y | 0.01 | —— |
| *Hypocrella bambusae* (Berk. et Br.) Sacc | Hypocreaceae | de ma | Fruit body | Alcohol maceration/oral | Amygdalitis |  |  | 0.02 | —— |
| *Calvatia gigantea* (Batsch ex Pers.) Lloyd | Lycoperdaceae | xiang ma ma qi | Fruit body | Crushed/external application | Wound |  | Y | 0.04 | —— |
| *Ganoderma lucidum* (Leyss. ex Fr. ) Karst. | Polyporaceae | —— | Fruit body | Alcohol maceration/oral | Insomnia |  | Y | 0.06 | —— |
| *Tremella aurantialba* Bandoni et Zang | Tremellaceae | —— | Fruit body | Cooked | Tumour |  | Y | 0.02 | —— |
| *Tricholoma matsutake* (Ito et Imai) Singer | Tricholomataceae | —— | Fruit body | Cooked | Hypoimmunity | VU | Y | 0.04 | —— |
| *Tuber melanosporum* Vittad. | Tuberaceae | —— | Fruit body | Alcohol maceration/oral | Hypoimmunity |  | Y | 0.03 | —— |
|  |  |  |  |  |  |  |  |  |  |
| **Lichens** |  |  |  |  |  |  |  |  |  |
| *Usnea longissima* Ach. | Parmeliaceae | —— | Whole plant | Decoction/oral | Wound |  |  | 0.01 | —— |
| *Thamnolia vermicularia* (Ach.) Asahina | Thamnoliaceae | —— | Whole plant | Decoction/oral | Asthma; Neurasthenia |  | Y | 0.01 | —— |

*Protection level: EN, endangered; VU, vulnerable; LC, least concern; (I) and (II), the protection level in national key preserved wild plants; II, the protection level in CITES.*
